# The emerging role of atrial strain assessed by cardiac MRI in different cardiovascular settings: an up-to-date review

**DOI:** 10.1007/s00330-022-08598-6

**Published:** 2022-04-22

**Authors:** Riccardo Cau, Pierpaolo Bassareo, Jasjit S. Suri, Gianluca Pontone, Luca Saba

**Affiliations:** 1 Department of Radiology, Azienda Ospedaliero Universitaria (A.O.U.), di Cagliari – Polo di Monserrato, s.s. 554 Monserrato, 09045 Cagliari, Italy; 2 University College of Dublin, Mater Misericordiae University Hospital and Our Lady’s Children’s Hospital, Crumlin, Dublin, Republic of Ireland; 3 Stroke Monitoring and Diagnosis Division, AtheroPoint(tm), Roseville, CA USA; 4 Department of Cardiology, Centro Cardiologico Monzino, IRCCS, Milan, Italy

**Keywords:** Atrial strain, Cardiac magnetic resonance, Feature tracking, Echocardiography, Advanced imaging

## Abstract

**Abstract:**

The left atrium (LA) has a crucial function in maintaining left ventricular filling, which is responsible for about one-third of all cardiac filling. A growing body of evidence shows that LA is involved in several cardiovascular diseases from a clinical and prognostic standpoint. LA enlargement has been recognized as a predictor of the outcomes of many diseases. However, LA enlargement itself does not explain the whole LA’s function during the cardiac cycle. For this reason, the recently proposed assessment of atrial strain at advanced cardiac magnetic resonance (CMR) enables the usual limitations of the sole LA volumetric measurement to be overcome. Moreover, the left atrial strain impairment might allow several cardiovascular diseases to be detected at an earlier stage. While traditional CMR has a central role in assessing LA volume and, through cine sequences, a marginal role in evaluating LA function, feature tracking at advanced CMR (CMR-FT) has been increasingly confirmed as a feasible and reproducible technique for assessing LA function through strain. In comparison to atrial function evaluations via speckle tracking echocardiography, CMR-FT has a higher spatial resolution, larger field of view, and better reproducibility. In this literature review on atrial strain analysis, we describe the strengths, limitations, recent applications, and promising developments of studying atrial function using CMR-FT in clinical practice.

**Key Points:**

• *The left atrium has a crucial function in maintaining left ventricular filling; left atrial size has been recognized as a predictor of the outcomes of many diseases*.

• *Left atrial strain has been confirmed as a marker of atrial functional status and demonstrated to be a sensitive tool in the subclinical phase of a disease*.

• *A comprehensive evaluation of the three phases of atrial function by CMR-FT demonstrates an impairment before the onset of atrial enlargement, thus helping clinicians in their decision-making and improving patient outcomes*.

**Supplementary Information:**

The online version contains supplementary material available at 10.1007/s00330-022-08598-6.

## Introduction

The function of the left atrium (LA) in modulating left ventricular fillings is very important and can be subdivided into three consecutive phases: (1) a reservoir for pulmonary veins during left ventricle (LV) systole; (2) a conduit for blood flow from pulmonary veins toward LV during LV early diastole; and (3) a booster pump to increase LV fillings during late diastole [1].

Over the past decade, LA size has been widely recognized as an essential marker of adverse cardiovascular events [2]. However, LA enlargement itself does not explain the whole LA function during the cardiac cycle. On the other hand, LA contractile function has been confirmed as a marker of LA functional status and a sensitive tool for detecting early-state diseases [3, 4]

Recently, strain analysis has been proposed as a tool to assess LA phasic function. LA volume and function are traditionally evaluated by echocardiography [4]. In this scenario, speckle tracking echocardiography (STE) was the first modality to assess atrial strain, but it has several limitations, including a suboptimal field of view in the setting of poor acoustic windows and high interobserver variability [5, 6]. Conversely, cardiac magnetic resonance (CMR) is a mainstay in the non-invasive assessment of LA volume and function [7]. Kowallick et al showed the potential of cardiac magnetic resonance feature tracking (CMR-FT) for assessing LA strain and strain rate (SR) parameters [8]. Several studies have demonstrated that LA deformation detected by CMR-FT can allow for an accurate and reproducible analysis of LA function [9, 10].

Another important aspect that needs to be analyzed is the evolving concept of atrial myopathy, first described by Zipes et al [11] as atrial remodeling following atrial fibrillation. Over the past 20 years, the concept of atrial myopathy has evolved with the current consensus definition: “Any complex of structural, architectural, contractile, or electrophysiological changes affecting the atria with the potential to produce clinically relevant manifestations.” [12] According to the EHRAS consensus’s definition, atrial myopathy can also be defined clinically as an abnormality in any of the three aspects of LA function [12].

This review summarizes the current understanding of atrial strain at CMR-FT and provides insights into its clinical application, including its prognostic role.

## Atrial strain principles and parameters

LV myocardial strain represents the percentage of longitudinal deformation of a myocardial segment, while strain rate (SR) measures the rate of change in strain during the cardiac cycle [13]. Three different LV myocardial strains are described: longitudinal (i.e., longitudinal shortening from the base to the apex), circumferential (i.e., shortening along the circular perimeter of the myocardium), and radial (the thickening and thinning of the myocardium) [13].

**Fig. 1:**
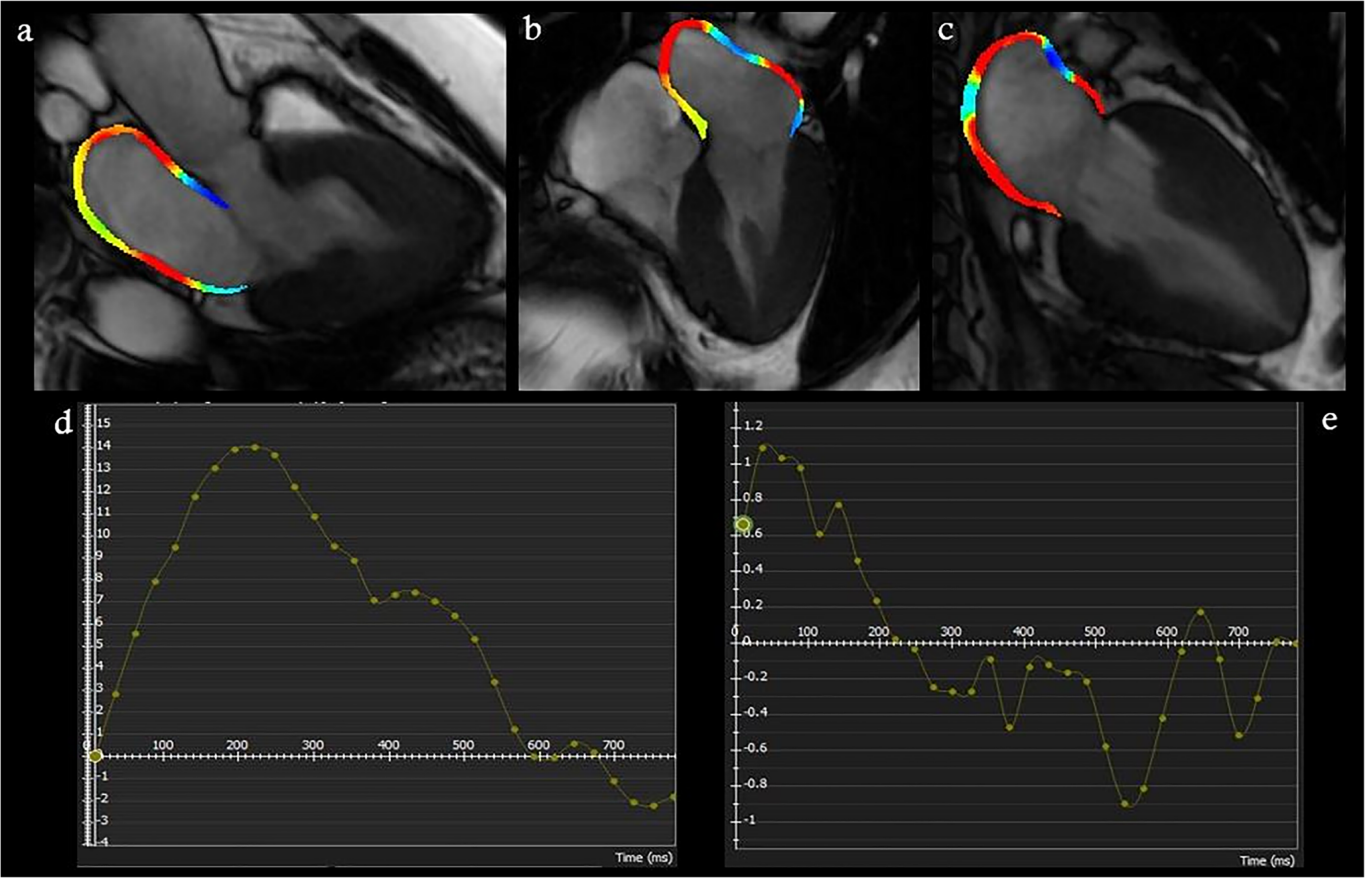
Representative images of total peak left atrial longitudinal strain by CMR-FT from (**a**) 3-, (**b**) 2-, and 4-chamber (**c**) view atrial strain using CMR-FT in a healthy subject. LA strain (**d**) and strain rate (**e**)

Meanwhile, due to the peculiar fibers’ orientations and the thinness of the atrial wall, only longitudinal strain is usually measured at the atrial level [1]. During the reservoir phase, the LA fills and stretches. It corresponds to a positive atrial strain that reaches its peak before mitral valve opening. Following that, in the conduit phase, there is a negative atrial strain due to passive LA emptying. Finally, in the booster phase, there is a second negative deflection corresponding to atrial systole [1, 14] (Fig. 1, Supplemental Video 1). LA is a dynamic chamber involving a significant interaction between LA function and LV performance during all cardiac cycles. The LA reservoir especially represents atrial relaxation and compliance influenced by the descent of the LV base during systole. LA conduit relies on atrial compliance during ventricular diastole, and it is closely related to LV relaxation and stiffness. Finally, LA booster reflects the intrinsic atrial contractility, which is modulated by the degree of venous return and LV diastolic compliance and pressure [1, 14, 15].

Truong et al investigated normal range values of LA strain and strain rate parameters using CMR-FT in 112 healthy volunteers. The authors reported a mean value of 39.13 ± 9.27, 25.15 ± 8.34, 13.99 ± 4.11, 1.93 ± 0.54, −2.13 ± 0.69, and −2.04 ± 0.61 for reservoir, conduit, and booster strain and strain rate parameters, respectively. In addition, they demonstrated that the reported strain values were comparable between genders, while LA booster and conduit function parameters changed significantly with age [16].

## Speckle tracking echocardiography vs. cardiac magnetic resonance

As previously mentioned, the most widely used modality for assessing LA phasic function is echocardiography [15]. STE is an echocardiography technique that tracks natural acoustic markers or “speckles” within the myocardium throughout the cardiac cycle [17]. Many studies have validated the feasibility and reproducibility of STE in studying LA function [18, 19]. STE has several strengths, such as its availability, reasonable cost, and analysis after the ultrasound images have been acquired and stored. Nonetheless, it also has its limitations, including the need for high-quality images, acquisition at a high frame rate, and intra-observer and inter-observer variability. For all these reasons, its clinical application remains somewhat limited [1, 3].

Nowadays, CMR is considered the gold standard imaging modality for evaluating the morphology and function of cardiac chambers, with LA volume and function included [16, 20–22]. It offers potential advantages in comparison with echocardiography, namely a wider field of view, high reproducibility, low intra- and inter-observer variability, and high tracking quality [23].

Unfortunately, CMR is limited by its long post-processing time, high cost, limited availability, and incompatibility with claustrophobia or the presence of metal devices inside the body (Table 1). To overcome the long post-processing time, Leng et al investigated the feasibility and effectiveness of a novel semi-automatic method for evaluating long-axis LA strain and strain rate using CMR compared to conventional FT strain analysis [24]. This proposed post-processing method does not require the total segmentation of the LA borders, but it does require the delineation of three distinct anatomical landmarks. The authors reported that semi-automatic LA strain analysis demonstrated excellent correlation and agreement compared to standard FT LA measurements, with a required mean time per subject of 85 ± 10 s (versus 190 ± 12 s using conventional FT analysis) [24].

**Table 1:** Non-invasive imaging in the assessment of LA function

Imaging modalities	Strengths	Limitations
Speckle tracking echocardiography	Safe Versatile Widely available technique High temporal resolution No radiation exposure or use of contrast	The need for high-quality image, acquisition at high frame rate. Anatomical plane restrictions Inter-observer variability
Cardiac magnetic resonance-feature tracking	Wide field of view High spatial resolution Excellent reproducibility Low intra- and inter-observer variability High tracking quality	Low availability Costs Intrinsic or extrinsic factors of the patient (claustrophobia, metallic implants, allergy, ability to hold breath, and arrhythmia) Long scan times

Atrial strain measurements using CMR-FT remain solely a research tool. One of the limitations for the widespread use of atrial strain analysis may be the limited data on inter-vendor differences. Pathan et al evaluated the inter-vendor comparison of all LA strain parameters using CMR-FT with two types of CMR post-processing software, namely Medis and CVI [25]. The two vendors showed significant differences in mean reservoir (limit of agreement −8.7 to 26.9%), conduit (limit of agreement −9.8%, 21.4%), and booster (limit of agreement −4.7 to 11.3%) in a Bland-Altman analysis. The authors concluded that observed inter-vendor normal reference ranges depend on which vendor is used to compute atrial strain; thus, they suggest cautious use during clinical applications concerning normal reference values [25].

Recently, CMR-FT has been used to quantify atrial deformation with a better accuracy than STE [26, 27]. In this respect, Truong et al demonstrated that, while good tracking was possible in only 91 out of 112 healthy subjects (81%) with STE analysis, tracking using CMR-FT was excellent for the whole cohort, with a good intra- and inter-observer agreement as well [16]. In CMR-FT, steady-state free precession (SSFP) cine CMR images are used and tracked offline to assess myocardial strain.

## Clinical application

Atrial strain analysis is progressively developing, owing to steady technological improvements. Thus, data are rapidly emerging regarding the potential role of LA strain analysis in different cardiovascular diseases (see Table 1) characterized by LA enlargement or stretching.

### Systemic disease

One of these illnesses is hypertension. In a study by Li et al on hypertensive patients with and without LV hypertrophy [28], LA strain parameters such as LA reservoir and conduit functions were significantly impaired independently of the presence of hypertrophy and in the absence of LA dilatation, thus suggesting that, in high blood pressure cases, strain abnormalities precede structural LA changes [28]. On the contrary, the authors reported that LA booster function was preserved in patients without LV hypertrophy [28].

LA abnormalities were also described in systemic diseases such as diabetes mellitus. In a study by Shao et al investigating atrial strain by CMR-FT in patients with type 2 diabetes [29], LA strains—especially LA global circumferential, LA global radial, and LA global longitudinal function—were reduced in comparison with healthy controls (*p* < 0.05 for all). The authors reported an improvement in LA strain after diuretic treatments, suggesting a protective effect (or a reversal) in the LA remodeling of these drugs [29]. In addition, LA strain was impaired even in patients with normal LV myocardial strain. These findings suggest that LA impairment may appear earlier than the LV strain abnormalities in the early stage of diabetes mellitus [29].

### Cardiomyopathy

Hinojar et al investigated LA contractile function using CMR-FT in seventy-five patients with hypertrophic cardiomyopathy (HCM) in comparison with the same number of healthy controls [3]. The authors also demonstrated impaired LA longitudinal function in the HCM patients with normal LA volume and LV filling pressure in comparison with the healthy control group, with high intra- and inter-observer agreements (*r* = 0.95 and *r* = 0.92, respectively) [3]. These findings are consistent with those reported by Yang et al, who found that LA reservoir and conduit impairment occur before LA enlargement in HCM patients [9]. These results imply that LA strain is more sensitive than LA volume in detecting LA involvement in HCM [3, 9].

Moreover, atrial strain using CMR-FT can help in the early detection of cardiac involvement in infiltrative cardiomyopathy due to Anderson-Fabry disease [30]. In the study by Bernardini et al, patients were stratified based on LV involvement. The main finding of the study was that the greater the LV involvement, the more reduced the LA global strain [30]. Additionally, atrial deformation showed a good correlation with native septal T1. The most atrial strain impairment was observed in patients with low T1 values, even in those without any LV hypertrophy or diastolic dysfunction. [30]

LA strain also provides useful information about patients with myocarditis [31, 32]. In a study on 30 patients with clinically proven myocarditis undergoing CMR, Dick et al demonstrated impairments in LA strain parameters during the reservoir and conduit phases [32]. The authors reported that LA early negative peak by SR proved to be the best predictor of acute myocarditis (AUC 0.80), with a sensitivity of 83% and a specificity of 80%, as well as an excellent intra- and inter-observer agreement (ICC = 0.91 and ICC = 0.91, respectively) [32]. Similar results were obtained by Doerner et al, who investigated the usefulness of CMR-FT strain (atrial deformation in particular) as a supplementary tool to the Lake Louise criteria in CMR-based diagnosis of myocarditis [31]. The authors showed that LA early negative peaks at SR and LV global longitudinal strain were very good tools (AUC = 0.72 and AUC = 0.69, respectively) in diagnosing myocarditis. Combining strain parameters and Lake Louise criteria increased the diagnostic accuracy (AUC = 0.77) [31].

### Ischemic heart disease

**Fig. 2:**
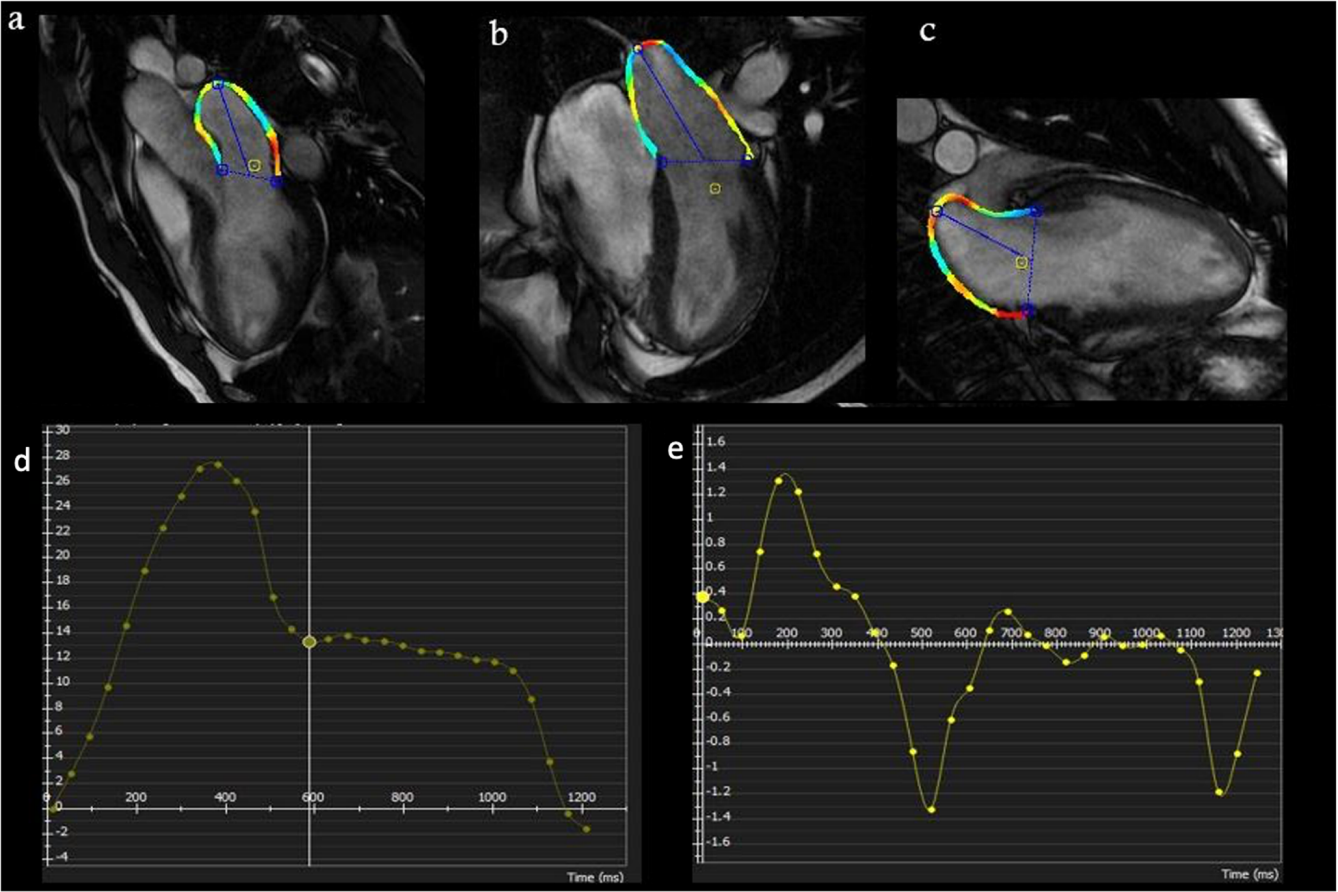
Representative images of total peak left atrial longitudinal strain by CMR-FT from 3 (**a**)-, 2 (**b**)-, and 4-chamber (**c**) view atrial strain using CMR-FT in post-myocardial infarction patients. LA strain (**d**) and strain rate (**e**)

Again, LA contractile function may be altered by ischemic heart disease. In this scenario, Kim et al evaluated the relationship between atrial strain and diastolic dysfunction in patients with myocardial infarction [33]. Their study demonstrated that atrial strain varied significantly according to the grade of LV diastolic dysfunction, with higher sensitivity than LA size [33]. Figure 2 and Supplemental Video 2 show an example of atrial strain in a patient with a history of previous myocardial infarction.

Lapinskas et al evaluated LA strain parameters using CMR-FT in patients with STEMI and secondary mitral regurgitation, demonstrating that all LA strain values—in particular, reservoir, conduit, and booster function strain—and strain rate parameters were significantly impaired in patients with secondary mitral regurgitation; only conduit strain rate parameters significantly increased in patients with mitral regurgitation [34].

### Atrial fibrillation

LA dysfunction is the rule in atrial fibrillation. Habibi et al demonstrated that, aside from LA booster function, all LA strain parameters at CMR-FT were significantly more impaired in patients with persistent atrial fibrillation than in healthy subjects, thus supporting the hypothesis of a progressive atrial dysfunction with time [27]. Another study reported that LA strain indices were predictive of the atrial fibrillation onset in patients with risk factors for stroke but without any history of atrial fibrillation, especially patients with LA booster below 17% who had a two-fold increased incidence of AF [35].

### Congenital heart disease

Atrial function was also studied in congenital heart disease cases [36, 37]. For example, in thirty-three patients with Fontan circulation, reservoir and conduit strain and strain rate parameters’ functions at CMR-FT were impaired in comparison with age-matched controls. Also, atrial strain was a strong predictor of a worsening in ventricular end-diastolic pressure, cardiac index, exercise capacity, liver stiffness, need for heart transplantation, ventricular assist device, or death [36]. Similar results were shown by Steinmetz et al in 30 patients with Ebstein’s anomaly who shared an impaired quantitative right heart atrio-ventricular deformation at CMR-FT, which is associated with heart failure severity, in terms of the NYHA class [37].

### Heart failure with preserved ejection fraction

LA mechanism (above and beyond LA size) was also evaluated in heart failure cases with preserved ejection fraction (HFpEF). von Roeder et al tested the role of LA strain function in HFpEF using CMR, demonstrating that LA reservoir and conduit strain were significantly lower in HFpEF (*p* = 0.04 and *p* < 0.01) and that LA conduit strain was the strongest predictor for exercise intolerance on multivariable regression analysis [38]. The authors demonstrated a functional LA remodeling in HFpEF, suggesting that LA conduit strain is an early marker of LA remodeling [38].

Moreover, LA reservoir and conduit strain are also promising prognostic markers in HFpEF. In 101 patients enrolled in a study by Chirinos et al, conduit strain, conduit strain rate, and reservoir strain were associated with increased risks of hospitalization and death [39].

## Prognostic role

LA size is linked with the outcomes of many cardiovascular diseases [4]. Guidelines recommend evaluating LA size as a marker of LV filling pressure [40]. LA strain is a promising marker and, likely, a better predictor of outcomes than LA size [4]. In this scenario, several studies have investigated the potential role of atrial strain parameters at CMR in envisaging cardiovascular adverse events [3, 33, 41, 42].

### Cardiomyopathy

For example, Hinojar et al evaluated the relationship between LA function and major cardiovascular outcomes—in particular, all-cause death and heart failure—by CMR-FT [3]. The authors found that impairment in longitudinal atrial strain is associated with increased mortality and heart failure onset (*p* = 0.04 and *p* = 0.002) [3]. This was particularly evident in the Kaplan-Meier analysis [3].

Increasing evidence indicates the significant role of atrial mechanism in the pathophysiology and prognosis of Takotsubo patients. Backhaus et al evaluated atrial involvement in patients with Takotsubo cardiomyopathy and its prognostic role using CMR-FT [41]. Regarding adverse clinical events, atrial strain parameters (in particular, atrial reservoir strain) revealed a greater AUC than LV ejection fraction (AUC = 0.69 vs. 0.59) and LA size (AUC = 0.69 vs. 0.62) [41].

### Ischemic heart disease

Other data have been used to evaluate LA function in predicting the outcomes after a myocardial infarction [33, 42], such as the onset of atrial fibrillation [33]. The authors showed that atrial longitudinal strain is superior to atrial size and LV diastolic dysfunction in envisaging the development of the arrhythmia [33]. In another study evaluating CMR in 321 post-myocardial infarction patients, Leng et al reported an impairment in left atrial reservoir strain and conduit strain [42]. The addition of atrial strain parameters to traditional MRI predictors of heart attack outcomes provided clinicians with greater prognostic accuracy when predicting major adverse cardiac events (0.75 vs. 0.66, *p* = 0.04) [42].

## Right atrial strain

**Fig. 3:**
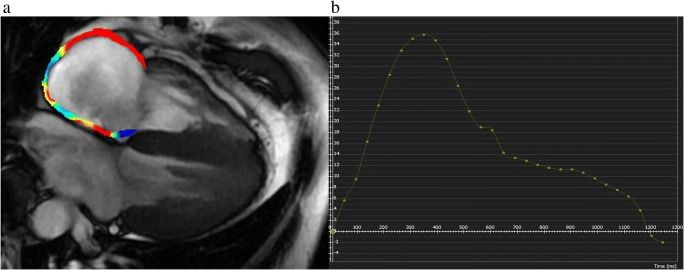
RA measurement by CMR-FT from 4-chamber (**a**) and LA strain curve (**b**) view in healthy subject

Both atria have important and different functions during the cardiac cycle. The right atrial (RA) is now being considered as more than a passive chamber and can be subdivided, like the LA strain, into three phases (reservoir, conduit, and booster) [43]. Measurements of RA deformation using STE are limited due to the retrosternal location of the RA, the presence of the caval venous system, and the very thin atrial wall [44, 45]. Conversely, CMR represents the reference standard for RA functional assessments (Fig. 3) [46]. Truong et al evaluated all phases of RA strain using CMR-FT in 61 healthy subjects, reporting mean values of 35.53 ± 14.35, 22.57 ± 11.08, 12.96 ± 6.36, 1.6 (1.3–2.2), −1.7 (−2.4 to −1.3), and −1.5 (−2.1 to −1.1) for reservoir, conduit and booster strain, and strain rate parameters, respectively [45].

### Pulmonary arterial hypertension

Many studies have displayed the crucial role of RA function in pulmonary arterial hypertension [47–49]. For instance, Hope et al evaluated the functional change in RA in patients with the same disease [47] and reported that RA longitudinal strain was impaired in comparison with healthy subjects. Also of interest, this RA parameter showed a significant difference between compensated and decompensated subgroups of pulmonary hypertension. In addition, RA longitudinal strain showed an excellent AUC (= 0.92) in identifying patients who are at risk of developing future adverse outcomes, with a sensitivity and specificity of 100% and 84.6%, respectively [47].

### Ischemic heart disease and cardiomyopathies

The usefulness of RA strain has also been evaluated in other clinical scenarios. Schuster et al investigated its application in 1235 patients after acute myocardial infarction [46]. RA reservoir and conduit parameters showed the most significance in predicting the onset of major adverse cardiovascular events and identifying the degree of heart failure [46]. Furthermore, Dick et al reported that both atria strain parameters were impaired in myocarditis cases [32].

### Heart failure with preserved ejection fraction

Studies have also evaluated RA function in HFpEF [50, 51]. Jain et al investigated RA strain (measured with CMR) among patients with HFpEF and evaluated the relationship between RA function and all-cause death. The authors found that RA reservoir and conduit function (but not booster function) were significant predictors of mortality, even after adjusting for confounders, suggesting that RA impairment may be an important cardiac marker in patients with or a risk of heart failure [50]. Also, von Roeder et al tested RA function in patients with HFpEF using CMR compared to invasive hemodynamic measurements. The authors reported that RA conduit function was lower between the control group and HFpEF (*p* < 0.01), while booster pump increased as compensation (*p* = 0.01) [51]. Table 2 summarizes the above-stated CMR-FT studies regarding the clinical and prognostic application of atrial strain.

**Table 2:** Previous studies regarding atrial strain using cardiac magnetic resonance feature tracking

Author and date published	Number (patients)	Type of study	Research	Results
Hinojar et al (2019)	75	Single-center observational study	Evaluated the LA contractile function using CMR-FT in HCM patients	Impairment of LA strain in HCM patients (*p* < 0.001) in comparison with control subjects with high intra- and inter-observer agreements (*r* = 0.95 and *r* = 0.92, respectively)
Yang et al (2020)	33	Single-center observational study	Investigated the LA function in patients with non-obstructive HCM using CMR-FT	Patients with non-obstructive HCM have LA reservoir, conduit and regional LA dysfunction (*p* = 0.01) when compared to healthy subject
Bernardini et al (2020)	45	Single-center observational study	Assessed LA function by CMR-FT in a population of patients with Anderson-Fabry diseases	Patients with Anderson-Fabry disease and a greater LV involvement showed a significantly reduced LA total strain. In addition, LA strain correlated well with the value of native septal T1
Von Roeder et al (2017)	22	Investigator-initiated, observational, single-center study (sub-study of STIFFMAN study)	Tested the role of LA strain function in HFpEF using CMR	LA reservoir and conduit strain were significantly lower in HFpEF (*p* = 0.04 and *p* < 0.01) and LA conduit strain was the strongest predictor for exercise intolerance on multivariable regression analysis
Chirinos et al (2018)	101	Prospective study	Evaluated the association of LA strain CMR with incident adverse cardiovascular events among subjects with or without heart failure (HF) at baseline	Conduit strain, conduit strain rate, and reservoir strain were associated with an increased risk of hospitalization and death
Jain et al (2019)	96	Prospective study	Investigated RA strain measured with CMR among patients with HFpEF and evaluated the relationship between RA function and all-cause death	RA reservoir and conduit function, but not booster function, were significant predictors of mortality, even after adjusting for confounders
von Roeder et al (2017)	24	Investigator initiated, observational, single-center study (sub-study of STIFFMAN study)	Tested RA function in patients with HFpEF using CMR	RA conduit function was lower between control group and HFpEF (*p* < 0.01), while booster pump was increased as compensation (*p* = 0.01)
Kim et al (2020)	257	Single-center observational study	Evaluated the diagnostic performance of atrial strain to stratify diastolic dysfunction in patients with myocardial infarction	Atrial strain was significantly different between diastolic dysfunction grades (*p* < 0.01), whereas LA size increased only with more advanced diastolic dysfunction
Lapinskas et al (2017)	20	Prospective pilot study	Evaluated LA strain parameters using CMR-FT in patients with STEMI and secondary mitral regurgitation	Reservoir, conduit, and booster function strain and strain rate parameters were significantly impaired in patients with secondary mitral regurgitation; only conduit strain rate parameters significantly increased in patients with mitral regurgitation
Shao et al (2020)	50	Single-center observational study	Investigated CMR-FT atrial strain in patients with type 2 diabetes mellitus	LA strain was impaired in patients with type 2 diabetes mellitus, even in patients with normal LV myocardial strain
Dick et al (2017)	30	Single-center observational study	Evaluated the diagnostic performance of LA and RA strain in detecting acute myocarditis	LA peak early negative SR proved to be the best predictor of acute myocarditis (AUC 0.80) with a sensitivity of 83% and specificity of 80%, with an excellent intra- and inter-observer agreement (ICC = 0.91 and ICC = 0.91, respectively)
Doerner et al (2018)	86	Single-center observational study	Evaluated the incremental diagnostic value of CMR-FT strain in patients with myocarditis	LA peak early negative SR and LV global longitudinal strain were the best predictors (AUC = 0.72 and AUC = 0.69, respectively) and a combination of strain parameters and Lake Louise criteria yielded higher diagnostic performance (AUC = 0.76%)
Li et al (2020)	87	Single-center observational study	Investigated CMR-FT LA strain index in hypertensive patients	LA strain parameters were significantly impaired in hypertensive patients without LA dilatation
Habibi et al (2015)	90	Single-center observational study	Assessed the role of LA function using CMR-FT in patients with atrial fibrillation	LA strain parameters were significantly lower in patients with persistent atrial fibrillation
Berteselen et al (2020)	203	Sub-study of the LOOP study (investigator-initiated, randomized controlled trial)	Evaluated whether atrial strain indices were able to predict atrial fibrillation in asymptomatic patients	LA strain indices were predictive of atrial fibrillation in patients with stroke risk factors but without history of atrial fibrillation
Critser et al (2021)	33	Multi-center observational study	Assessed atrial function in Fontan patients using cardiac MRI	Atrial function was impaired in Fontan patients in comparison with age-matched control and atrial pump strain was a predictor of primary outcome
Steinmetz et al (2018)	30	Single-center case-control study	Evaluated atrial and ventricular myocardial deformation in Ebstein’s anomaly	LA function was impaired and atrial booster function showed a correlation with NYHA class
Hinojar et al (2019)	75	Single-center observational study	Evaluated the relationship between LA function using CMR-FT and major cardiovascular outcomes in HCM patients	Impaired longitudinal atrial strain is associated with cardiovascular mortality and heart failure. In particular, Kaplan-Meier curves showed that patients with an impaired atrial strain experienced a significantly higher rate of all cause-death and heart failure (*p* = 0.04 and *p* = 0.002)
Kim et al (2020)	257	Single-center observational study	Evaluated the diagnostic performance of atrial strain to predict atrial fibrillation in patients with myocardial infarction	Left atrial strain improved prognostic for predicting atrial fibrillation post-myocardial infarction in comparison with atrial geometry
Leng et al (2020)	321	Prospective, multicenter study	Investigated LA strain parameters as long-term predictors in myocardial infarction	Left atrial reservoir strain and conduit strain after myocardial infarction were impaired after myocardial infarction patients. Strain parameters provided incremental prognostic value in comparison with the traditional MRI risk factors (0.75 vs. 0.66, *p* = 0.04)
Backhaus et al (2019)	152	Multi-center observational study	Assessed the diagnostic and prognostic potential of atrial strain parameter in Takotsubo patients	Atrial strain parameters overriding outcome predictor, such as left ventricular ejection fraction and LA size (AUC = 0.69 vs. AUC = 0.59; AUC = 0.69 vs. AUC = 0.62, respectively)

## Conclusion

Atrial strain is an emerging parameter with multiple confirmed important clinical and prognostic applications. STE at advanced echocardiography is the most widely used modality for measuring LA strain, given the thinness of the atrial wall. However, CMR-FT, as an alternative modality, offers higher spatial resolution, superior contrast, and greater reproducibility under the same settings. A comprehensive evaluation of the three phases of atrial function by CMR-FT can demonstrate impairments before the onset of atrial enlargement, thus informing clinicians’ decisions and improving patient outcomes.

## Supplementary Information

ESM 1 (AVI 9654 kb)

ESM 2 (AVI 9676 kb)

## Abbreviations

CMR: Cardiac magnetic resonance
FT: Feature tracking
HFpEF: Heart failure with preserved ejection fraction
LA: Left atrium or left atrial
LV: Left ventricle
RA: Right atrium or right atrial
SSFP: Steady-state free precession
ST: Strain rate
STE: Speckle tracking echocardiography

## Declarations

### Guarantor

The scientific guarantor of this publication is Luca Saba.

### Statistics and biometry

No complex statistical methods were necessary for this paper.

### Methodology

• performed at one institution
